# Long-term care based on person-centered care for the older adults in rural Spain: the rural care project

**DOI:** 10.3389/fpubh.2025.1601427

**Published:** 2025-09-22

**Authors:** Elena Betegón, Jairo Rodríguez-Medina, Clara González-Sanguino, María Jesús Irurtia

**Affiliations:** ^1^Department of Psychology, University of Valladolid, Valladolid, Spain; ^2^Department of Pedagogy, University of Valladolid, Valladolid, Spain

**Keywords:** healthcare, quality of life (QoL), long-term care, older adults, person-centered care (PCC), rural care, World Health Organization Quality of Life (WHOQOL-BREF) scale

## Abstract

**Introduction and objective:**

The Rural Care Project is a social and healthcare initiative designed for older adults living in deep-rural areas of the Autonomous Community of Castile and Leon (Spain) who require long-term care (LTC). This intervention program promotes Person-Centered Care (PCC) and active aging to improve quality of life (QoL). The aim of this study is to evaluate the project's effectiveness on QoL by way of a quasi-experimental study.

**Methods:**

A total of 416 Spanish participants were divided into three groups: (a) experimental group (*N* = 102) made up of adults residing in deep-rural areas and receiving targeted home-based support; (b) control: residential care group (*N* = 170) with people receiving extensive formal care; (c) control: at home (*N* = 144), consisting of older adults with clinically identified dependency, disability, or chronic illness, who remained in their homes in rural areas and relied mainly on informal support (family, neighbors, or self-management), receiving little or no formal LTC services. The intervention spanned 18–20 months and included social and psychological support, coordination of care services, and periodic assessments by trained professionals. QoL was assessed pre- and post-intervention using the “World Health Organization Quality of Life” (WHOQOL-BREF) scale. Data were analyzed using repeated measures ANOVA, with *post hoc* tests to explore group differences. Power analysis confirmed adequate sample size to detect medium effects (α = 0.05, power = 0.80, effect size = 0.5).

**Results:**

Participants reported high satisfaction with personal relationships and housing. The experimental group showed significant improvements in physical and psychological health post-intervention, with moderate and small effect sizes, respectively. Improvements in social relationships and environmental context were limited, appearing mainly in the Control: at home group.

**Discussion and conclusion:**

The findings provide evidence that the Rural Care program effectively improves key dimensions of QoL among older adults in deep-rural areas. Recommendations include implementing policy reforms to promote home-based LTC grounded in PCC principles. Prioritizing tailored support to enhance physical health and reduce medical dependency are critical outcomes that should be emphasized. Although the program did not produce significant effects on environmental context and social relationships, observed trends suggest potential benefits if future interventions are expanded to comprehensively address these areas. Thus, future programs should adopt a multifaceted approach, integrating strategies for environmental enhancements and promoting both formal and informal social interactions to empower older adults in decision-making processes.

## Introduction

The aging of the population, particularly in rural areas, is one of the most pressing challenges currently faced by European societies. While increased life expectancy is considered a triumph—reflecting advances in healthcare, environment, and social development (1)—it also requires innovative and sustainable long-term care (LTC) models that promote autonomy, quality of life, and social inclusion among older adults. In rural contexts, where demographic aging is more pronounced, the limited availability and uneven implementation of person-centered, home-based LTC models present a significant barrier to adequately addressing the needs of dependent older individuals (2). This study addresses that gap by evaluating the piloting of the Rural Care project—an integrated, Person-Centered Care (PCC) initiative implemented in the rural region of Castile and Leon (Spain). The project's effectiveness is assessed through a quasi-experimental pre-post study comparing outcomes among participants in the Rural Care program and two control groups: one composed of older adults living at home in rural areas, with some degree of dependency, disability, or chronic illness, who relied mainly on informal care or self-management and had little or no access to formal LTC services (Control: at home), and another of care home residents (Control: residential care group).

In 2021, 22.8% of the European Union (EU) population was 65 years of age or older and it is estimated that this figure will reach 29.1% in 2050 (3). As far as rural areas are concerned, all of them have aging populations (4). In Spain in 2022, 20.08% of the population was 65 or older and it is expected that this figure will reach 30.38% in 2050 (5), and the Autonomous Community of Castile and Leon is the worst affected areas, where the greatest demographic aging of Spain's rural areas can be observed. In this region, 47% of local council areas have more than half of their population over the age of 65, with this figure reaching 80% in some councils (6).

The aging of the population in rural areas has significant healthcare implications, including less accessibility to the healthcare system and a greater prevalence of chronic illnesses (7–9) among with significant challenges in terms of emotional and social welfare. Loneliness and social isolation are particularly common due to the lack of infrastructures and services and a lower degree of mobility and social interaction (10–13). This can increase the risk of depression, anxiety and other mental health problems, ultimately leading to a lower quality of life (QoL) among older adults in these areas (14–17).

Long-term care (LTC) is defined as the system of informal and formal care aimed at helping people who cannot completely look after themselves to maintain the best possible QoL in accordance with their preferences (18). This approach is normally employed in combination with PCC, in which social and healthcare professionals work together with users to identify the distinctiveness and uniqueness of each individual (2, 19, 20). Different studies have shown how older adults receiving PPC-based LTC have a better QoL compared with traditional methods of care (21–25). It has also been demonstrated that they are more satisfied with the care they receive (26) and have a greater degree of general satisfaction (27–30).

In Spain the types of LTC available to the older adults include (31): (a) home-based care: enables older adults to receive care in their homes. Services may include personal care (bathing, dressing), meal preparation, and companionship; (b) day centers: Offer social and recreational activities during the day, allowing family caregivers to work or take a break; and (c) care homes (residential facilities): institutional settings where older adults live and receive 24-h care, catering to various needs, including medical, social, and personal care. Various professionals play crucial roles within this system: nurses (provide direct care to LTC recipients, assist with daily activities, administer medications, and monitor health conditions), physicians (oversee medical aspects, diagnose health issues, and prescribe treatments) and social workers (assess social needs, coordinate services, and provide emotional support to LTC recipients and their families).

Some cases of the application of LTC and PPC for older adults in Spain have shown good results in terms of perceived satisfaction, QoL and levels of depression and behavioral problems (32, 33). In spite of the well-known advantages of LTC and PCC, the degree to which they have been implemented in Spain is mixed, with an increase in the number of informal carers (mainly family members) with no specialized training in home care who tend to see a negative effect on their own QoL (34, 35). Regarding the structure of the Spanish healthcare system for LTC, Spain operates a tax-based LTC financing system managed at the regional level, funded by national, regional, and local resources. The system encompasses both community-based and institutional care services.

To achieve the implementation of these national coverages, regional regulation is necessary in each of the autonomous communities. In the region of Castile and Leon, the Individual Care Program *Programa Individual de Atención* (PIA) is currently implemented to provide tailored benefits based on the specific dependency needs of each individual (36) hoping to have benefits. This dependency is classified into three levels: (a) Grade III or High Dependency: when a person requires assistance for multiple basic daily activities several times a day; (b) Grade II or Severe Dependency: when a person needs help with several basic daily activities and need extensive assistance for personal autonomy; and (c) Grade I or Moderate Dependency: when a person requires assistance with several basic daily activities at least once a day or has intermittent or limited needs for support in personal autonomy. Regarding the service catalog, options include dependency prevention, promotion of personal autonomy, telecare, home assistance, day and night care centers, as well as residential centers. These services are managed through the public network of social services of the Autonomous Community, which includes duly accredited public and privately contracted entities.

In terms of economic benefits, options include financial aid for home care, personal assistance, and financial benefits linked to service acquisition. The latter is intended to cover professional services included in the individual's PIA, when adequate public services are not available or when appropriate service choices or benefits are not selected according to the dependency situation.

Rural Care (37) arose as a social healthcare initiative in the region of Castile and Leon to respond to these needs. The programme has been implemented in rural areas with an aging population in need of integrated care. The main objective of the project is to promote PCC and active aging, with the aim of improving the QoL of the older adults and their carers. It is carried out in partnership with different institutions and receives funding from the European Social Fund Plus (ESF+) and the regional health ministry of the Autonomous Community of Castile and Leon (38).

The aim of the present study is to evaluate the piloting of the Rural Care project, analyzing the results of its implementation via a quasi-experimental pre-post study comparing the results of the Rural Care participant group with the two control groups (Control: at home, consisting of people living at home, and Control: residential care group, care home residents).

## Method

### Participants

The selection of the study groups for the pilot project was conducted in collaboration with the Social Services Management of the regional health ministry of the Autonomous Community of Castile and Leon, which facilitated access to the participant population through its social work staff. This institution is responsible for designing and implementing regional social policy plans and strategies in coordination with public and private entities within the region's Social Services System (36).

Based on the needs and characteristics of the population observed by this public body over the years, 60% of the beneficiaries were women, reflecting the demographic profile of the selected area, which meets the criteria for a Deep-Rural Area. This classification is characterized by low income, significant aging, a predominance of women, and population dispersion.

A total of 416 older adults aged 60–101 years (M = 82.32, SD = 11.56) participated in the study. Of these, 293 (59.55 %) were women and 199 (40.45%) men. Participants were divided into one experimental and two control groups.

- The experimental group (experimental) consisted of 102 individuals receiving home-based support as part of the Rural Care program and living in their community.
- The control group receiving care at home (control: at home; *N* = 144), older adults with clinically identified LTC needs (dependency, disability, or chronic illness) who remained in their rural homes. They relied primarily on informal care (provided by family, neighbors, or through self-management) and had little or no access to formal home-care services.
- The control group of care homes (control: residential care group; *N* = 170), consisted of participants residing in publicly-run care homes.

This distinction is particularly relevant within the Spanish LTC model, where formal services in rural areas are scarce and care depends largely on informal networks. To better capture this variability, participants were also classified using the Home at Risk (HR) system, which stratifies households by degree of dependency and availability of caregivers. The HR classification comprises four levels:

- HR1 corresponds to households with individuals with Grade I dependency with viable caregivers and adequate care.
- HR2 includes households with individuals with Grade I or Grade II dependency accompanied either by adequate care or by fragile caregivers, understood as those who, due to age, physical or mental health limitations, emotional burden, or lack of resources, are unable to provide stable or sufficient care.
- HR3 comprises households with individuals with Grade II dependency with viable caregivers but with insufficient care.
- HR4 encompasses single-person households with individuals with Grade III dependency or with fragile caregivers, as defined above.

This classification system enabled the identification and prioritization of households requiring more intensive support and resources within the regional social services system, ensuring a targeted and structured intervention through the Rural Care program.

Power analysis confirmed adequate sample size to detect medium effects (α = 0.05, power = 0.80, effect size = 0.5), and it was consistent to other studies (39). The inclusion criteria for groups experimental and the control: at home group were as follows: (a) living in a deep-rural area; (b) having some kind of dependence, disability or chronic illness; (c) requiring LTC; and (d) remaining at home as their primary residence. For the Control: residential care group, the inclusion criteria were the same with the exception of their usual place of residence being a publicly-run care home. Given the limited number of care homes in rural areas, it was necessary to extend the geographical scope to include urban care homes to meet the inclusion criteria. However, since these were all publicly managed institutions, they operated under the same regulatory standards and care protocols, regardless of their geographic location.

### Procedure

The study was developed within the framework of the Programme for Employment and Social Innovation, “EaSI” 2014–2020, in the call for proposals entitled *Call for proposals on social innovation and national reforms (Long-Term Care)*, Grant Agreement VS/2020/0290.

A quasi-experimental design was employed to evaluate the effectiveness of the Rural Care program in improving the quality of life among older adults living in deep-rural areas. Participants had previously been assigned to one of three study groups—one experimental and two control groups—based on their existing care conditions and place of residence. As random assignment was not feasible due to contextual and logistical constraints, this study followed a non-randomized, pre-post design, which is a common approach in long-term care intervention research (39, 40).

The intervention was implemented in the experimental group through the Rural Care program, which offered structured home-based support tailored to individual needs. This included assessments, coordination of services, and psychosocial support over an 18–20-month period. In contrast, the control groups continued with their usual care: either extensive formal care in residential facilities or minimal to no formal care at home. The project began in October 2020, with the first assessment being carried out in May 2021 and the last in July 2022. Information about the programme had been disseminated among social healthcare professionals in the field of geriatric care through the regional government of Castile and Leon, SACYL (the regional health service), the provincial government, rural councils and centers and services for innovation in education and healthcare.

Participants were recruited following referral from geriatric care professionals, who served as a link, providing information (either verbally or via an explanatory leaflet) about the existence of the programme to future participants or their immediate family. Subsequently, social workers from the Social Services Department of Castile and Leon conducted follow-up phone calls to confirm participation, provide further information, and schedule home visits. In the case of the Control: residential care group, contact was made through the geriatric professionals directly caring for the participants in the different care homes.

After selection and detailed explanation of the study procedures, and once informed consent and a commitment to participate in the project had been signed, an initial assessment was conducted. The same procedure was performed after a period of 18–20 months, depending on the pace with which the project was carried out. Data collection for both the control and experimental groups was synchronized to ensure consistency in the timing of assessments.

Assessments of all the groups were conducted by two expert assessors (psychologists), specifically trained in the assessment protocol, which included a structured interview with sociodemographic questions and self-administered questionnaires. Each assessment lasted ~45 min.

Within the experimental group, the HR classification system set the boundaries of potential support agreements with participants, which were reviewed by coordinators and case managers from the Social Services Management. These limits could be adjusted with the proposal of the case coordinator and approval from the project coordinator, provided that the amount contributed to each household did not exceed 50% of the total cost of home adaptations and technical aids. For loans of technical aids, funding was provided at 100%, with no cost to the user.

All HR levels were assigned, at different percentages (36):

- Home adaptation, technical aids and advanced telecare.
- Training and support for non-professional caregivers.
- Personalized social participation program.
- Case manager and reference professional.
- Community relations with a minimum of two actions per day.
- Personal care, as needed, up to 24 h in periods of 2–5 days per month.
- Home healthcare, both scheduled and emergency, according to the service portfolio and chronic and palliative care processes.

Training, selection, and evaluation of the support actions were carried out by staff from the Social Services User Support System (*Sistema de Atención a Usuarios de Servicios Sociales*, SAUSS). The remaining direct care services were provided by “Fundación Personas,” a non-profit organization with extensive experience in supporting people with disabilities and other LTC services. Currently, this organization supports 4,000 people in both in urban and rural areas of the region.

Finally, the financial guidelines were (37): for HR4, a maximum of €4,500 per household was financed; for HR3, a maximum of €2,700 per household was financed; for HR2, a maximum of €1,540 per household was financed; and for HR1, a maximum of €680 per household was financed.

In the control groups, the existing LTC services were maintained as they were prior to the start of the project. It is important to note that the conventional LTC programs differ significantly from the model proposed by the Rural Care Project, which prioritizes maintaining the user in their home, adapting services as their needs evolve. The latter prioritizes keeping the user within their own home, adapting the provided aids and services as their needs evolve. This approach contrasts with conventional LTC programs, where users are often moved to care homes when their support needs become extensive or specific. In such facilities, the sense of belonging and connection to their social environment is not as strong as it is in their own home.

### Human ethics and consent to participate declarations

In all the procedures, the ethical standards of the institutions, the criteria of the National Research Committee, and the international criteria of the American Psychological Association (APA) (41) and the 1964 Declaration of Helsinki (42) (as well as their subsequent amendments or similar ethical rules), were followed. Within these ethical principles for research involving human subjects, the confidentiality of the data and the pursuit of the benefit of the participants are ensured. The study was approved by the ethics committee and by the European Commission in accordance with Grant Agreement VS/2020/0290.

All participants (or their legal guardians, when applicable) were informed about the aims and procedures of the study and provided written informed consent prior to participation, in line with ethical guidelines.

### Variables and tools

Sociodemographic data were collected by asking participants about their sex (male or female) and age. To measure QoL, the “World Health Organization Quality of Life” (WHOQOL-BREF) (43) scale was employing in its Spanish version, adapted by Lucas-Carrasco (44–46). The questionnaire had good psychometric properties both in the original (Cronbach alphas demonstrate good internal consistency for the facets with a range of 0.65–0.93) (43) and Spanish version (ω = 0.89) (45). The WHOQOL-BREF consists of 24 items rated on a Likert-type scale from 1 (completely dissatisfied) to 5 (completely satisfied), focusing on the participants' level of satisfaction regarding various aspects of their QoL in the previous 2 weeks. The scale covers four factors:

a) Physical health (Factor 1—seven items): Referring to pain and discomfort experienced, perceived fatigue and energy levels, and sleep and rest, ranging from the individual's control over their pain to their reluctance or enthusiasm to carry out everyday tasks and sleep problems.
b) Psychological health (Factor 2—six items): Including the presence of positive and negative feelings, aspects relating to thoughts, learning, memory and concentration, self-esteem, body image and appearance.
c) Social relationships (Factor 3—three items): Including company, love and support in personal relationships, as well as the social support provided by friends and family and sexual activity.
d) Environment (Factor 4—eight items); Relating to the degree of physical safety perceived by the person, their place of residence, economic resources, access to social and healthcare, participation in and opportunities for recreational and leisure activities, access to transport and the characteristics of their physical environment in relation to pollution or climate.

### Data analysis

A descriptive analysis was conducted of the responses to each block of the questionnaire. Before conducting the main analyses, multivariate outliers were examined using Mahalanobis *D*^2^ distances (with a significance threshold of α = 0.001) and Guttman error analysis, in order to detect atypical response patterns. These procedures helped ensure data quality and robustness in the subsequent statistical analyses. Then, the data were analyzed via a repeated measures ANOVA in order to verify the differences between groups and phases. Thus, a mixed design was proposed of repeated measures with an intra-subject factor (the moment of the assessment) and an inter-subject factor (the group). The intra-subject factor has two levels (pretest and posttest), while the inter-subject factor has three. The scores obtained in each of the sub-scales of the WHOQOL-BREF QoL questionnaire were taken as dependent variables. In order to verify the differences between groups, a *post hoc* analysis was performed.

To estimate the necessary sample size, the *pwr* package in R was employed. A significance level of 0.05 was established, along with a statistical power of 0.80 and an effect size of 0.5. This effect size (*d* = 0.5) was selected based on Cohen's (47) conventions for medium effects, as no directly comparable studies reporting effect sizes in similar rural intervention contexts were available.

The calculation of the statistical power indicates that, with a sample size of 30 participants, there would be a probability of 0.80 of detecting an effect size of 0.5. A sample size of 30 participants is sufficient for detecting an effect size of 0.5 with a statistical power of 0.80.

## Results

### Descriptive analysis

Although the WHOQOL-BREF scoring guidelines (43, 46) do not recommend interpreting individual item responses, item-level results are reported here for exploratory and descriptive purposes. These results aim to offer more detailed insights into participants' perceptions in specific areas of quality of life, particularly relevant in rural intervention contexts. Findings should be interpreted with caution and do not replace domain-level analyses.

First of all, Table 1 shows the relative frequencies obtained in each of the response options for all of the items and the accumulated frequencies and relative frequencies for the higher (four and five) and lower (one and two) values of the scale. In the second phase relating to health (item 2), 28.21% answered that they were dissatisfied or extremely dissatisfied, whereas 35% considered themselves to be quite or extremely satisfied.

**Table 1:** Relative frequencies for each response option.

**Item**	**Likert scale**										**Percentage of responses**			
	**1 (Not at all)**		**2 (Not much)**		**3 (Moderately)**		**4 (A great deal)**		**5 (Completely)**		**% 1–2**		**% 4–5**	
	***Pre***	***Post***	***Pre***	***Post***	***Pre***	***Post***	***Pre***	***Post***	***Pre***	***Post***	***Pre***	***Post***	***Pre***	***Post***
**Physical health**														
3r	9.56	3.18	29.41	22.49	13.73	20.29	17.89	19.8	29.41	34.23	38.97	25.67	47.3	54.03
4r	17.4	8.31	0.49	27.14	20.83	32.76	26.23	31.3	8.33	8.31	44.61	27.63	34.56	39.61
10	9.07	3.67	24.75	26.65	34.56	39.61	26.72	25.67	4.9	4.4	33.82	30.32	31.62	30.07
15	14.46	6.11	31.86	30.32	22.79	33.74	15.2	20.29	15.69	9.54	46.32	36.43	30.88	29.83
16	9.56	9.05	18.38	19.8	20.1	22.98	43.63	31.3	8.33	16.87	27.94	28.85	51.96	48.17
17	9.56	2.44	22.79	27.14	31.13	30.56	31.13	28.85	5.39	11	32.35	29.58	36.52	39.85
18	12.75	3.18	19.36	30.07	40.44	32.76	24.51	25.43	2.94	8.56	32.11	33.25	27.45	33.99
**Psychological health**														
5	22.79	14.67	29.41	30.32	27.21	34.96	19.36	19.32	1.23	0.73	52.21	44.99	20.59	20.05
6	11.27	10.02	18.63	13.69	32.84	37.16	35.78	34.23	1.47	4.89	29.9	23.72	37.25	39.12
7	5.15	5.13	18.87	18.83	28.92	30.07	39.46	29.58	7.6	16.38	24.02	23.96	47.06	45.97
11	6.62	2.44	13.48	13.2	39.71	55.01	37.01	27.63	3.19	1.71	20.1	15.65	40.2	29.34
19	6.13	3.18	13.48	11	27.94	35.7	48.77	45.72	3.68	4.4	19.61	14.18	52.45	50.12
26	5.15	1.22	20.59	18.83	26.23	30.56	30.15	29.83	17.89	19.56	25.74	20.05	48.04	49.39
**Social relationships**														
20	1.23	0.24	5.15	3.67	20.83	29.1	58.09	60.64	14.71	6.36	6.37	3.91	72.79	66.99
21	35.29	9.54	24.75	26.41	38.48	63.33	1.47	0.73	0	0	60.05	35.94	1.47	0.73
22	2.94	2.44	24.02	25.18	35.54	39.36	27.94	30.32	9.56	2.69	26.96	27.63	37.5	33.01
**Environmental context**														
8	4.41	0	14.71	14.67	24.26	24.21	53.68	51.1	2.94	10.02	19.12	14.67	56.62	61.12
9	0.49	0	12.5	5.38	58.33	69.19	15.44	24.69	13.24	0.73	12.99	5.38	28.68	25.43
12	4.41	2.44	30.39	21.27	44.85	50.61	18.38	23.96	1.96	1.71	34.8	23.72	20.34	25.67
13	6.37	4.16	15.2	16.63	23.28	30.81	46.08	31.54	9.07	16.87	21.57	20.78	55.15	48.41
14	33.33	25.67	32.11	31.78	24.02	31.05	9.31	11.25	1.23	0.24	65.44	57.46	10.54	11.49
23	1.47	1.47	3.43	4.16	23.77	23.96	55.88	61.37	15.44	9.05	4.9	5.62	71.32	70.42
24	2.94	3.67	9.31	10.76	44.12	40.34	40.2	41.56	3.43	3.67	12.25	14.43	43.63	45.23
25	16.42	3.91	22.3	24.94	52.94	67.73	6.86	3.42	1.47	0	38.73	28.85	8.33	3.42

Items are part of the WHOQOL-BREF questionnaire and rated on a five-point Likert scale (1 = not at all, 5 = completely). “Pre” and “Post” refer to baseline and follow-up assessments. Percentages represent the proportion of responses in each category. The columns “% 1–2” and “% 4–5” indicate the percentage of responses grouped at the lower and upper ends of the scale. Items marked with “r” were reverse-coded according to WHOQOL-BREF guidelines.

More than 70% of the participants considered themselves to be quite or extremely satisfied with their personal relationships (item 20) and with the conditions of their homes (item 23), both in phase 1 and phase 2. Around 50% claimed to feel quite safe (item eight—phase 1 = 51.04%; phase 2 = 56.41%; Table 1).

As far as the two questions of a global nature are concerned (items 1 and 2), the results were divided. In the pretest, around 25% of the participants classified their QoL (item 1) as *normal*, while 37% claimed it was quite or extremely good and another 37% said it was average or extremely bad. In relation to their general state of health (item 2), around 25% classified it as *normal*, with 39% stating that it was good or extremely good and 36%, bad or extremely bad. In the second phase, the results were similar, although for item 1 the percentage of people claiming to have a normal QoL went from 25 to 35%, those who stated that their QoL was extremely bad or average went from 37 to 25 and 39.61% classified it as quite or extremely good compared to the 37% who did so in the pretest. Likewise, as far as the participants' satisfaction with their general health is concerned, around 35% claimed it was *normal* (vs. 25% in the pretest) and another 30% claimed they were quite or extremely dissatisfied (vs. 36% in the pretest).

### Multivariate outliers

Multivariate outliers were analyzed using Mahalanobis *D*^2^ distances, applying a significance threshold of α = 0.001 (48). Fourteen observations exceeded the critical value, indicating significant multivariate outliers. The highest *D*^2^ value observed was 228.11. These results are described to support the robustness of the sample prior to the main analyses.

Subsequently, the number of Guttman errors was calculated for each of the observations with the aim of identifying atypical response patterns. The average number of errors was 270.19 (SD = 138.77), in line with the criterion proposed by Zijlstra et al. (49) and by Hubert and Vandervieren (50) for asymmetric distributions. The critical value is 582.5, thus 11 cases with atypical response patterns were identified.

### Physical health

Table 2 shows the results relating to physical health on a scale of 0–100. Differences between groups can be observed in the initial assessment, which would later be verified to see whether they were significant. On the other hand, an increase can be observed in the mean score relating to physical health in the experimental group (Pre = 41.28; Post = 53.85) and in the Control: residential care group (Pre = 50.97; Post = 56.10) compared with a decrease in the physical health score in the Control: at home group (Pre = 54.60; Pre = 53.87).

**Table 2:** Means and standard deviations for WHOQOL-BREF domains.

**Domain**	**Group**	**Assessment**					
		**Pre**		**Post**		**Overall**	
		***M***	***SD***	***M***	***SD***	***M***	***SD***
Physical health	Experimental	41.28	20.88	53.85	17.14	47.57	20.07
	Control: residential care group	50.97	20.23	56.10	16.99	53.53	18.82
	Control: at home	54.60	17.94	53.87	15.72	54.23	16.84
	Marginal	50.08	20.15	54.64	16.51		
Psychological health	Experimental	47.75	16.61	53.64	15.55	50.69	16.32
	Control: residential care group	56.02	15.06	57.18	15.41	56.60	15.22
	Control: at home	50.78	17.84	52.08	15.34	51.43	16.62
	Marginal	51.85	16.90	54.23	15.54		
Social relationships	Experimental	50.16	14.46	51.88	13.82	51.02	14.13
	Control: residential care group	53.76	12.96	54.28	12.62	54.02	12.77
	Experimental	50.16	14.46	51.88	13.82	51.02	14.13
	Control: at home	46.72	15.99	51.37	12.19	49.04	14.39
	Marginal	50.00	14.91	52.50	12.79		
Environmental context	Experimental	51.32	9.86	52.27	9.17	51.79	9.51
	Control: residential care group	54.01	10.32	55.97	10.31	54.99	10.35
	Control: at home	50.02	10.49	52.44	9.52	51.23	10.07
	Marginal	51.72	10.40	53.62	9.85		

M and SD represent mean and standard deviation, respectively. “Overall” refers to the average (mean and standard deviation) calculated across all groups at each assessment time point (Pre and Post).

An approximately normal distribution can be observed for the physical health scores. The ANOVA shows significant differences among the groups in the pretest for physical health [*F* (2.413) = 15.11, *p* < 0.01, η^2^]. The *post hoc* tests show that the experimental group obtained significantly lower scores on this sub-scale in the pretest than the Control: residential care group (*t* = −3.84, *p* < 0.01) and the Control: at home (*t* = −5.45, *p* < 0.01). No statistically significant differences were observed between the Control: residential care group and Control: at homes (*t* = 1.65, *p* = 0.23).

**Figure 1:**
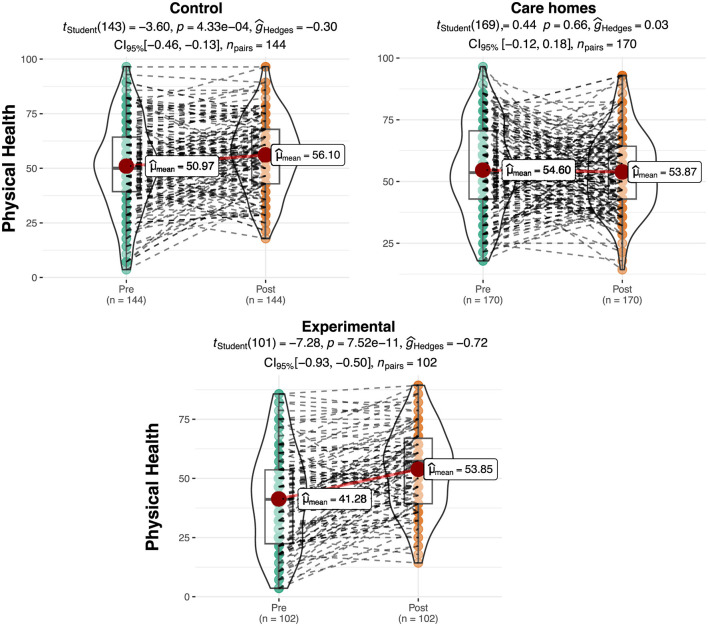
Differences in scores for physical health per group and phase. The plots show score density; red dots indicate group means and dashed lines represent individual trajectories.

However, no significant differences were observed between groups in the posttest data analysis [*F* (2.413) = 0.87, *p* = 0.42]. The repeated measures ANOVA produced a significant interaction effect result between the group (Experimental, Control: at home, Control: residential care) and the phase [Pre vs. Post; *F* (2.409) = 24.41, *p* < 0.01, η^2^ = 0.021]. Figure 1 shows a graphic representation of the result. It can be observed that there is a significant effect on the experimental (*p* < 0.001, *d* = 0.72) and control groups (*p* < 0.001, *d* = 0.3), with moderate and small effect sizes, respectively. However, there is no significant effect on the Control: at home group (*p* = 0.66).

### Psychological health

Table 2 shows the results relating to psychological health on a scale of 0–100. In the initial assessment, differences can be observed among the groups, which would subsequently be verified to see if they were significant. As with the case of physical health, a greater increase in the mean score for psychological health was observed in the experimental group (Pre = 47.75; Post = 53.64) compared to a moderate increase for the control groups (Pre Control: residential care = 56.02; Pre Control: at home = 50.78; Post Control: residential care = 57.18; Post Control: at home = 52.08).

**Figure 2:**
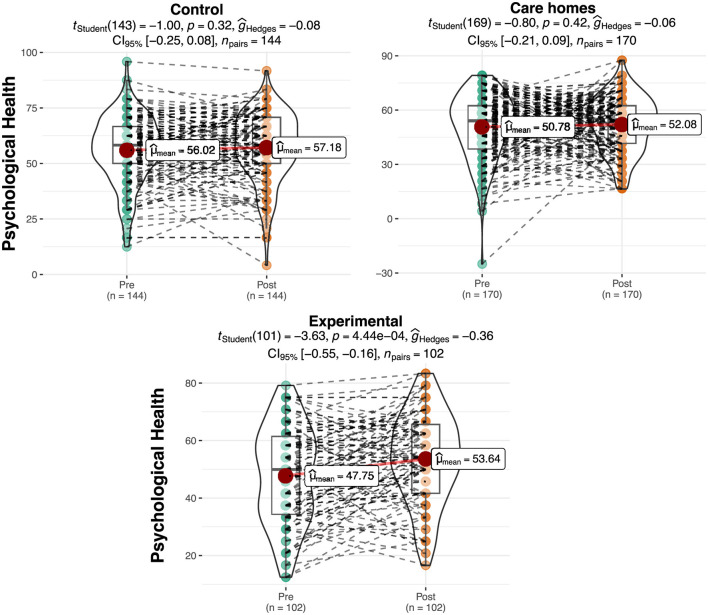
Differences in the scores for psychological health per group and phase. The plots show score density; red dots indicate group means and dashed lines represent individual trajectories.

The ANOVA shows significant differences between the groups in the pretest for psychological health [*F* (2.413) = 7.98, *p* < 0.01, η^2^ = 0.038]. The *post hoc* tests show that the experimental group obtained significantly lower scores in this sub-scale in the pretest than the Control: residential care group (*p* < 0.01). No significant differences were observed between the experimental group and the Control: at home group in the pretest. On the other hand, significant differences were observed between the Control: at home, and Control: residential care group groups in the analysis of the posttest data [*F* (2.413) = 4.35, *p* < 0.05, η^2^ = 0.021]. In this regard, the Control: at home obtained significantly lower scores than the Control: residential care group (*p* < 0.05). The repeated measures ANOVA produced a significant interaction effect result between the groups and the phase [Pre vs. Post; *F* (2.409) = 3.93, *p* < 0.05, η^2^ = 0.004]. Figure 2 shows a graphic representation of the result. It can be seen that the increase in the mean score in the experimental group between the pretest and the posttest is statistically significant (M_pre_ = 47.75, M_post_ = 53.64, *t* = 3.63, *p* < 0.001, *d* = 0.360) with a small effect size. On the other hand, the increase in the mean score in the Control: residential care group (M_pre_ = 56.02, M_post_ = 57.18, *t* = 0.997, *p* = 0.32) and Control: at home (M_pre_ = 50.78, M_post_ = 52.08, *t* = 0.802, *p* = 0.424) groups is not.

### Social relationships

Table 2 shows the results relating to the quality of social relationships on a scale of 0–100. Differences were observed among the groups in the initial assessment, which would later be verified to see whether they were significant. A slight increase can be observed in the mean score for social relationships in the experimental group (Pre = 50.16; Post = 51.88) and in the Control: residential care group (Pre = 53.76; Post = 54.28) and Control: at home (Pre = 46.72; Post = 51.37) groups.

**Figure 3:**
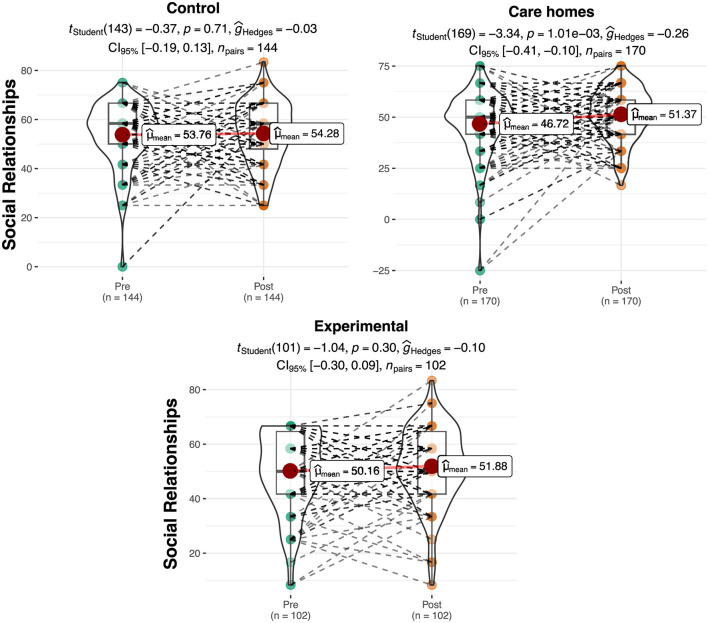
Differences in scores for social relationships per group and phase. The plots show score density; red dots indicate group means and dashed lines represent individual trajectories.

The ANOVA showed significant differences among the groups in the pretest in terms of the quality of their social relationships [*F* (2.413) = 9.05, *p* < 0.01, η^2^ = 0.04]. In this case, the *post hoc* tests show that the Control: at home obtained significantly lower scores in this sub-scale in the pretest than the control group (*t* = −4.25, *p* < 0.01). On the other hand, no significant differences can be observed among the groups in the analysis of the posttest data [*F* (2.413) = 2.9, *p* = 0.11]. The repeated measures ANOVA did not produce significant interaction effect results between the groups and the phase [Pre vs. Post; *F* (2.409) = 2.46, *p* = 0.087; Figure 3]. However, the Control: at home improved its score significantly for social relationships between the pretest and the posttest (*t* = 3.34, *p* < 0.001, *d* = 0.26) with a small effect size.

### Environmental context

Table 2 shows the results relating to the quality of environmental context on a scale of 0 to 100. Differences were observed in the initial assessment among the groups, which would later be verified to see if they were significant. A slight increase can be observed in the mean score for the quality of environmental context in the experimental group (Pre = 51.32; Post = 9.86) and in the Control: residential care group and Control: at homes (Pre Control: residential care group = 54.01; Pre Control: at home = 50.02; Post Control: residential care group = 55.97; Post Control: at home = 53.62).

The ANOVA showed significant differences among the groups in the pretest for the quality of their social relationships [*F* (2.413) = 5.60, *p* < 0.01, η^2^ = 0.028]. In this case, the *post hoc* tests show that the Control: at home obtained significantly lower scores in this sub-scale in the pretest than the Control: residential care group (*t* = −3.43, *p* < 0.01). No significant differences can be observed between the experimental group and the Control: at homes in the pretest. In the posttest, significant differences were observed among the groups [*F* (2.413) = 6.43, *p* < 0.01, η^2^ = 0.03], specifically between the Control: residential care group and the experimental group (*t* = −2.94, *p* < 0.01) and between the Control: residential care group and the Control: at home (*t* = −3.20, *p* < 0.01).

**Figure 4:**
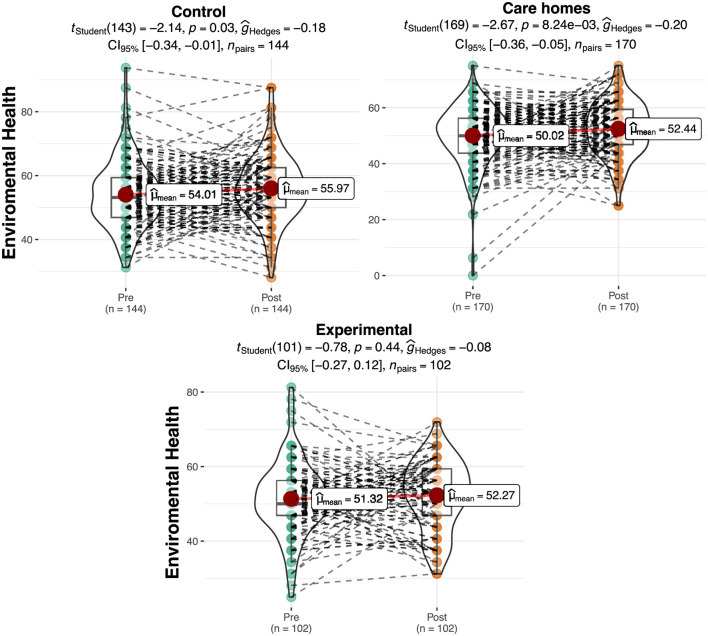
Differences in scores for environmental context per group and phase. The plots show score density; red dots indicate group means and dashed lines represent individual trajectories.

The repeated measures ANOVA did not produce significant results for interaction effects between the group and the phase [Pre vs. Post; *F* (2.409) = 0.500, *p* = 0.61]. However, the Control: at home significantly improved its score for environmental context between the pretest and the posttest (*t* = 3.34, *p* < 0.001, *d* = 0.26) with a small effect size (Figure 4).

## Discussion

The present study examines the impact of Rural Care, a care model based on LTC and PPC for older adults in deep-rural areas, analyzing its effects on the QoL of the participants. The total average age of the sample studied is 82.32 years, with a higher proportion of women (59.55%).

The results obtained after the application of the Rural Care programme showed, in general terms, improvement in the experimental group in the posttest compared to the pretest for QoL, physical and psychological health. In terms of physical health, the experimental group showed a significant increase in the posttest in comparison of people living at home and in care homes. These results are in line with prior studies, in which improvements in QoL have been found following the application of these types of programmes. In their systematic review and meta-analysis, Kim and Park (51) found that person-centered interventions improved depression and QoL among older adults with dementia. Replicating these results, the systematic review by Chenoweth et al. (52) showed that, after a year of application, LTC had significant effects in improving the QoL of older adults with dementia. Other more recent studies have shown similar results, such as Howard et al. (53) and Chen et al. (54), who found that PPC and LTC improve the QoL of older adults.

As for the dimensions of environmental context and social relationships, the results did not show significant effects of the programme, although trends of improvement were observed in the data, both for the experimental group and for the care home residents. This is coherent as Rural Care only contemplated intervention in interpersonal relationships if it had been stated as a need for the person (not in all cases), in accordance with the recommendation of employing a hierarchy of needs in the intervention. This aligns with other research, such as that conducted by Delgado (55), where significant relationships between self-perceived health and QoL, as well as life satisfaction, were observed, but no such relationships between these same variables and social support. Additionally, as Brownie and Nancarrow (56) point out,

“the introduction of person-centered care is not always incorporated within a wider “hierarchy of needs” structure, where safety and physiological need are met before moving onto higher level needs” (p.1).

Furthermore, the increase in the improvement of social relationships in people living in care homes can also be explained by the fact that the structure of such institutions facilitates social contact, which is often a complex issue in isolated rural areas (57, 58). In short, in rural areas, older adults experience unwanted loneliness due to multiple factors such as the loss of their social circle, the migration of young people and family members to other areas with better services, and the lack of resources (59). Other research (60–62) associates this subjective feeling of loneliness with a higher risk of becoming physically frail, diminished affection toward their emotional bonds, and an increased risk of depression—key elements as a correlation has been found between loneliness and QoL (63).

There was also a positive trend in terms of environmental context for all of the groups, although no significant differences were found. This may be due to the intervention needs of the individual people, which may be covered by both formal and informal care, as pointed out in the previous section (24, 30, 53). Other studies examining LCT and PCC, such as that of Marventano et al. (23), have found an improvement in QoL associated to functional independence, health status and informal social interaction (family, friends and/or neighbors). Furthermore, people receiving LTC at home showed an improvement in intra-psychological skills, such as resilience (59), particularly due to improvements in the infrastructure of the home and its maintenance through homecare services (25). One example is the research by Geigl et al. (64), who show that higher income levels, an internal locus of control, regular exercise, and strong social support are all associated with better physical and mental wellbeing. Consequently, they advocate designing integrated—health and social—interventions tailored to at-risk profiles (advanced age, low social support, or an external locus of control) to reduce inequalities and enhance the quality of life of older adults.

In accordance with all of the above, it can be concluded that interventions should be multifaceted, encompassing environmental improvements and an increase in formal and informal social interactions. This implies changes in terms of LTC programmes and their management, focusing them on the empowerment of older adults in decision-making based on a PCC model (56). However, it should be stressed that there is a need for more research in this field in order to determine the results of this type of programme due to the diversity of results and the consequences of aging.

As Van Malderen et al. (39) point out in their review, high-quality research that comprehensively addresses quality of life in long-term care remains scarce. Multidimensional interventions—those that act simultaneously on physical health, the environment, and social relationships—are the most promising, but they require consensus on which quality-of-life dimensions to include, standardization of measurement tools, and the design of rigorous studies to support evidence-based recommendations. An illustrative example comes from Siqeca et al. (65), who, using an ecological approach with nearly 9,000 older adults in Switzerland, demonstrated how individual determinants (physical health, polypharmacy, educational level), social factors (support network, participation in activities), and macro-level elements (type of health insurance) interact to shape health-related quality of life. Their findings underscore the need for truly integrated interventions capable of simultaneously addressing all these levels to maximize impact.

The present study has certain limitations which imply that the results should be taken with caution. First, due to the nature of the target population, sampling and recruitment proved complicated, making it extremely difficult to achieve the necessary number of participants via random assignment. The lack of randomization between the control and experimental groups is a clear limitation; however, access to rural areas is complex, and in many cases, the characteristics of these regions hinder project dissemination and participant recruitment. Although the project was well received, it was necessary to broaden the inclusion criteria for the Control: residential care group.

Another limitation concerning group assignment is that the experimental group had substantially lower pre-intervention scores in physical and psychological health compared to the control groups in community housing and residential care. Additionally, it showed lower scores in social and environmental dimensions relative to the community housing group, which may have biased the comparisons between groups.

Spasova et al. (66) note that the challenges of the LTC system are common across European countries, but that, in the vast majority, these services lack proper integration between social and health aspects and are organized at different administrative levels: local, regional, and national. Local services are the most affected due to the user-to-service ratio, that is, “the law of supply and demand.” Another important point raised by IŽdonaite-MedŽiuniene and Preikšaitiene (67) is that, although there is concern for quality of life, older adults often lack the training and resources needed to turn their intentions into sustainable healthy habits. Therefore, it would be necessary to include specific training in addition to the material supports or social resources provided by programs like Rural Care.

On the other hand, it should be noted that, although the assessments were carried out rigorously by trained assessors using valid, reliable and widely-used tools, QoL is an extremely broad and complex construct, which is difficult to represent with a single tool. In addition, there are very few tools adapted to the older adults, and some items seemed irrelevant to the study population (e.g., assessment of sexual life).

Additionally, it should be noted that, although the WHOQOL-BREF is designed to provide valid scores at the domain level and not for interpretation of individual items, this study included some item-level results for exploratory purposes. This decision aimed to offer more detailed insights into specific aspects of participants' quality of life, particularly relevant in rural contexts. However, we acknowledge that this approach does not strictly follow the instrument's methodological recommendations, and item-level findings should be interpreted with caution and limited to their descriptive value. However, from this perspective, the exploratory review by Arias-Casais et al. (40) warns that comprehensive, high-quality, and homogeneous research remains scarce, and that many initiatives focus on a single determinant when quality of life is, by definition, multidimensional. Furthermore, they note that approaches combining exercise, nutrition, and person-centered care are particularly promising, but require standardization of measures and consensus on quality-of-life dimensions; at the same time, they stress the need to include low-resource interventions, harmonize definitions across different contexts, and conduct more robust effectiveness evaluations to underpin global recommendations.

Furthermore, it is possible that certain biases inherent to self-report questionnaires, such as social desirability, may have also affected the results. On the other hand, it should be noted that the generalizability of the results may be limited due to the specific sociocultural environment where the project was carried out. Finally, although the pre-post longitudinal data have been shown, the project is still ongoing, so another limitation of the study is the lack of long-term follow-up of the results of the intervention. This is a clear line of follow-up research in the future, hoping that a longer-term consolidation of the results and impact of the programme can be shown.

## Conclusion

Rural Care highlights the physical and psychological QoL benefits of a person-centered program tailored to rural settings. Providing care that addresses the specific needs of older people in rural areas—while tackling challenges related to accessibility, affordability, quality and sustainability in depopulated rural regions— is essential for promoting dignified aging and safeguarding wellbeing and QoL. The project demonstrates the usefulness of multilevel partnership in care delivery, involving public and private actors and the coordination of social and health services at local, regional and national levels.

Additionally, the continuation of the Rural Care project presents a valuable opportunity to conduct follow-up assessments to evaluate the long-term effects on QoL and the sustainability of observed benefits. Addressing these gaps will yield crucial insights for policymakers aiming to implement integrated care models across diverse socio-cultural and geographical contexts.

## Data Availability

The datasets presented in this article are not readily available because the dataset is subject to several restrictions due to its nature and origin. It contains sensitive health-related information from a vulnerable population, requiring strict confidentiality and ethical considerations. Additionally, as the data is part of a project involving public entities, its access and use are governed by specific legal and regulatory frameworks. Any use of the dataset must comply with applicable data protection laws and ethical guidelines to ensure the privacy and wellbeing of the individuals involved. Requests to access the datasets should be directed to elena.betegon@uva.es.
